# Evidence That a RecQ Helicase Slows Senescence by Resolving Recombining Telomeres 

**DOI:** 10.1371/journal.pbio.0050160

**Published:** 2007-06-05

**Authors:** Julia Y Lee, Marina Kozak, Joel D Martin, Erin Pennock, F. Brad Johnson

**Affiliations:** Department of Pathology and Laboratory Medicine, University of Pennsylvania School of Medicine, Philadelphia, Pennsylvania, United States of America; Rockefeller University, United States of America

## Abstract

RecQ helicases, including Saccharomyces cerevisiae Sgs1p and the human Werner syndrome protein, are important for telomere maintenance in cells lacking telomerase activity. How maintenance is accomplished is only partly understood, although there is evidence that RecQ helicases function in telomere replication and recombination. Here we use two-dimensional gel electrophoresis (2DGE) and telomere sequence analysis to explore why cells lacking telomerase and Sgs1p (*tlc1 sgs1* mutants) senesce more rapidly than *tlc1* mutants with functional Sgs1p. We find that apparent X-shaped structures accumulate at telomeres in senescing *tlc1 sgs1* mutants in a *RAD52-* and *RAD53*-dependent fashion. The X-structures are neither Holliday junctions nor convergent replication forks, but instead may be recombination intermediates related to hemicatenanes. Direct sequencing of examples of telomere I-L in senescing cells reveals a reduced recombination frequency in *tlc1 sgs1* compared with *tlc1* mutants, indicating that Sgs1p is needed for *tlc1* mutants to complete telomere recombination. The reduction in recombinants is most prominent at longer telomeres, consistent with a requirement for Sgs1p to generate viable progeny following telomere recombination. We therefore suggest that Sgs1p may be required for efficient resolution of telomere recombination intermediates, and that resolution failure contributes to the premature senescence of *tlc1 sgs1* mutants.

## Author Summary

Because telomeres are situated at the ends of chromosomes, they are both essential for chromosome integrity and particularly susceptible to processes that lead to loss of their own DNA sequences. The enzyme telomerase can counter these losses, but there are also other means of telomere maintenance, some of which depend on DNA recombination. The RecQ family of DNA helicases process DNA recombination intermediates and also help ensure telomere integrity, but the relationship between these activities is poorly understood. Family members include yeast Sgs1p and human WRN and BLM, which are deficient in the Werner premature aging syndrome and the Bloom cancer predisposition syndrome, respectively. We have found that the telomeres of yeast cells lacking both telomerase and Sgs1p accumulate structures that resemble recombination intermediates. Further, we provide evidence that the inability of cells lacking Sgs1p to process these telomere recombination intermediates leads to the premature arrest of cell division. We predict that similar defects in the processing of recombination intermediates may contribute to telomere defects in human Werner and Bloom syndrome cells.

## Abstract 3

Yeast cells lacking the RecQ helicase Sgs1p show an accumulation of telomere recombination intermediates associated with premature senescence.

## Introduction

Telomeres are critical for genome stability and normal cell physiology because they cap the ends of chromosomes; if uncapped, telomeres behave as DNA breaks and thus elicit damage responses and are subject to nucleolytic degradation and recombination [1,2]. Capping depends on telomere architecture, which is mediated by chromatin factors, and on telomere length. The enzyme telomerase can counteract the shortening of telomeres that accompanies DNA replication or DNA damage, but dividing cells lacking sufficient telomerase can develop critically short, uncapped telomeres that signal cell cycle arrest (cell senescence) or death. Some cells bypass these barriers by up-regulating telomerase expression and thus elongating telomeres. In other cases, bypass involves the use of recombination to maintain telomere length. Examples of the latter case are so-called “survivors” of telomerase deletion in Saccharomyces cerevisiae and alternative lengthening of telomeres (ALT) cells in mammals [3,4].

A growing number of proteins are recognized as participating in telomere maintenance [2]. Among these are members of the RecQ family of DNA helicases [5], including the human Werner syndrome (WS) and Bloom syndrome proteins (WRN and BLM, respectively) and S. cerevisiae Sgs1p. Deficiencies in these helicases lead to genome instability caused by defects in recombinational repair of DNA damage, replication fork stability, and checkpoint signaling, and can lead to the premature onset of cancer and age-related pathologies [5,6]. The precise mechanisms by which RecQ helicases help maintain telomeres are not yet clear, but there is evidence that they are important for telomere replication, repair, and recombination [7–18]. A well-characterized function of RecQ helicases throughout the genome is the regulation of homologous recombination, by which they facilitate resolution of recombination intermediates and perhaps avoid the initiation of inappropriate recombination events [5]. Yeast survivors of telomerase deletion and mammalian ALT cells are two settings in which RecQ helicases are important in recombination-dependent telomere maintenance. For example, Sgs1p is required for emergence of type II survivors, which depend on recombination among telomere repeat sequences [15–17]; the Schizosaccharomyces pombe RecQ homolog SPAC212.11 similarly facilitates survivor emergence [7], and WRN regulates the generation of ALT cells from murine telomerase knockout cells [19]. In addition to their roles in survivors and in ALT cells, RecQ helicases function in telomere maintenance in primary cells that have little or no telomerase activity. For example, human WS fibroblasts suffer occasional complete loss of a telomere, which occurs preferentially at the guanine-rich telomere strand, which is replicated by lagging-strand synthesis [11,20]. These loss events presumably contribute to the premature senescence of cultured WS cells and their arrest at longer mean telomere lengths than control cells [21]; even though the shortening of most telomeres may be normal in WS cells, the increased frequency of occasional and critically shortened telomeres could signal senescence. Further, mutations in *Wrn* or *Blm* synergize with short telomeres in telomerase knockout mice to cause several degenerative pathologies, indicating that the helicases play important roles in telomere maintenance [10,12]. And in yeast, although *sgs1* mutants maintain telomeres of normal length in the presence of telomerase, *tlc1 sgs1* mutants senesce faster than *tlc1* mutants [15,17]. The rapid senescence of *tlc1 sgs1* mutants is due to an increased propensity of cells lacking Sgs1p to suffer G2/M arrest at a given average extent of telomere shortening; this suggests a role for Sgs1p in the repair of rare, critically shortened telomeres that would otherwise be repairable by telomerase if it were active. We recently described evidence that Sgs1p uses recombination functions to maintain telomeres during senescence, similar to its role in survivors of senescence. In particular, Sgs1p was shown to function in a *RAD52*-dependent pathway during senescence, and to use known recombination functions, including helicase activity and cooperation with topoisomerase III [22]. Here we use more direct methods, including 2DGE and sequence analysis of telomeres from a single chromosome end to investigate telomere maintenance in *tlc1* and *tlc1 sgs1* mutants. Our findings indicate that Sgs1p is required for efficient resolution of recombining telomeres during senescence. Moreover, telomere sequence analysis suggests that cells that have entered into telomere recombination require Sgs1p to give rise to viable progeny. These findings emphasize the importance of RecQ helicase recombination functions in telomere maintenance during senescence, and suggest that failure of recombination functions contributes to rapid senescence in cells with mutations in RecQ helicases.

## Results

**Figure 1:**
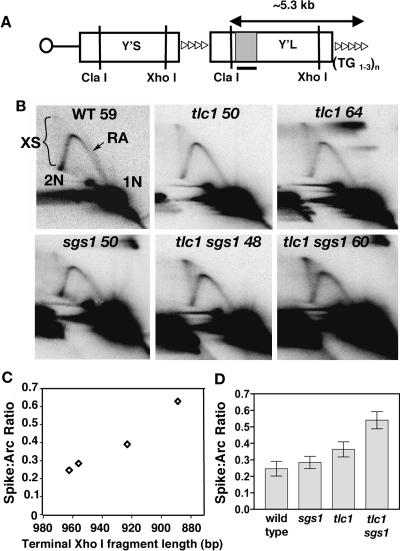
Telomere Replication and X Structures in Senescing *tlc1* and *tlc1 sgs1* Mutants (A) An example of a typical telomere with two tandem subtelomeric Y′ elements. Most of the Y′-containing telomeres in our yeast strain have the long version of Y′ (Y′L) at the terminal position immediately followed by TG_1–3_ sequence (arrowheads), and most internal Y′ elements are the short version (Y′S). The locations of ClaI restriction sites (the line representing three tightly clustered sites), XhoI restriction sites, and the Y′L-specific probe (bar below gray Y′L-specific sequence) are indicated. (B) Genomic DNA was harvested from log-phase cultures of the indicated strains at the PD indicated (upper right of each panel), digested with ClaI, separated by 2DGE, blotted, and probed for Y′L. Y-shaped replication arcs (RA), X-structures (XS), nonreplicating (1N), and nearly fully replicated (2N) species are indicated. (C) X-structure levels from one clone of *tlc1* cells at four different PD from the time of loss of telomerase are charted as a function of telomere terminal restriction fragment (TRF) length; PDs are 49.6, 56.3, 62.7, and 67.8 from left to right. TRF lengths were determined by Southern blotting of XhoI-digested genomic DNA probed for the Y′ 3′ end, and X-structure levels were normalized to replication arc levels (spike:arc ratio). (D) Comparison of X-structure levels (normalized to replication arc levels) in independent cultures each of wild-type (*n* = 5), *sgs1* (*n* = 5), *tlc1* (*n* = 10), and *tlc1 sgs1* (*n* = 10) mutants. Mean PDs for *tlc1* and *tlc1 sgs1* cultures were 57 and 52, respectively. The mean and standard errors are shown; *tlc1 sgs1* levels were 50% higher than *tlc1* levels (*p* < 0.025).

Nondenaturing 2DGE and Southern analysis were used to examine possible changes in telomere replication or recombination caused by *sgs1* and *tlc1* mutations. Each S. cerevisiae telomere contains from zero to four tandem copies of a subtelomeric element called Y′ [23]. Thus Y′ elements may be “terminal” and followed only by telomere repeat sequences, or “internal” and followed by at least one Y′ element prior to the telomere terminus. There are two Y′ element size classes (Y′ long [Y′L] and Y′ short [Y′S]), with the longer, Y′L, containing a 1.5-kilobase (Kb) element absent from Y′S (Figure 1A). To simplify analysis, and because in our yeast strain Y′L is the predominant class and most Y′L elements occupy a terminal position, a Y′L-specific probe was used to visualize telomeres. Replication of Y′ and telomere repeat DNA was observed predominantly in a Y-arc pattern indicating that most replication of the terminal Y′ elements derives from origins centromeric to the terminal Y′ elements (Figure 1B). No change in the replication pattern was observed in the *sgs1* mutant, consistent with a previous report [24], nor in senescing *tlc1* or *tlc1 sgs1* mutants (Figure 1B). In particular, there was no accumulation of replication forks in the telomere repeat DNA near the end of the Y arc (2N spot), indicating that, at least under typical circumstances, *SGS1* is not required for fork progression through telomere repeat sequences. A “spike” extending upward from the 2N spot was visible (Figure 1B); species running in this position have been attributed to X-shaped structures, including convergent replication forks and various recombination intermediates (see below) [25–27]. The intensity of the spike (normalized to the intensity of the replication arc) did not increase significantly in *sgs1* compared to wild-type cells (Figure 1B and 1D). However, the spike intensity increased significantly as telomeres shortened in the *tlc1* mutant (Figure 1B and 1C). This is consistent with observations in Kluyveromyces lactis that telomere recombination rates increase at short telomeres [28]. Moreover, the spike intensity was 50% higher in *tlc1 sgs1* compared to *tlc1* cells at equivalent population doublings (PD) after loss of telomerase (*p <* 0.025; Figure 1D). Similar results were also obtained with a probe that detected all Y′ elements (Supplementary Figure 1 in Protocol S1).

**Figure 2:**
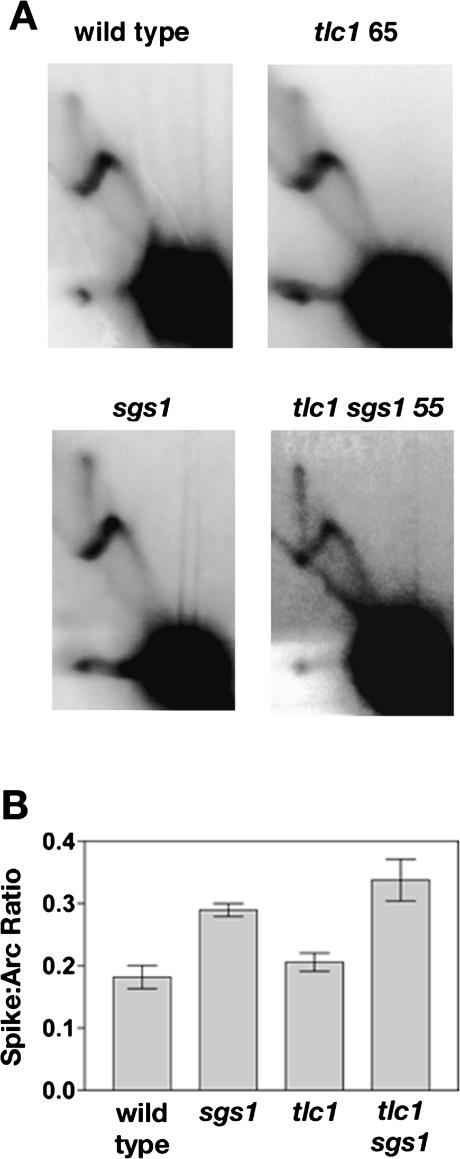
X-Structure Levels in the Ribosomal DNA in Wild-Type, *sgs1, tlc1,* and *tlc1 sgs1* Cells (A) Genomic DNA was isolated from log-phase cultures of the indicated genotypes and PDs, and was digested with SnaBI, separated by 2DGE, and visualized with a probe against the 18S rDNA region. (B) rDNA X-structure levels were quantified in comparison to replication arc levels (spike:arc ratio) in samples obtained from three independent cultures each of wild-type, and *sgs1, tlc1,* and *tlc1 sgs1* mutant cells; means and standard errors are shown. rDNA X-structure levels were elevated by *sgs1* mutation (wild type vs. *sgs1: p* = 0.007; *tlc1* vs. *tlc1 sgs1 p* = 0.023), but were not elevated by *tlc1* mutation.

To address the specificity of the increased X-structures at telomeres, X-structure levels were examined in the ribosomal DNA (rDNA). Although rDNA X-structures were elevated by *sgs1* mutation, consistent with elevated rDNA recombination and higher X-structure levels in *sgs1* mutants in earlier reports [29,30], telomere shortening caused by *tlc1* mutation caused no increase in levels (Figure 2), indicating specific accumulation at telomeres with senescence. X-structures at several genomic loci have been found to accumulate in late S or G2/M phases of the cell cycle. Because senescent telomerase mutants similarly accumulate in the G2/M phase of the cell cycle [15,31,32], this raised the possibility that elevated telomere X-structures in senescent cells might simply reflect a cell cycle effect. However, the lack of increased rDNA X-structures with senescence indicates that this is not the case. Similarly, the lack of increased telomere X-structures in *sgs1* mutants indicates that perturbations in cell cycle progression reported by some investigators in these mutants [29,33–35] do not explain the increases that occur during senescence. Rather, elevated telomere X-structures are somehow related specifically to telomere shortening, and Sgs1p plays a role in attenuating the accumulation of telomere X-structures in this context. Such a role of Sgs1p might be to prevent the formation of telomere recombination intermediates or to facilitate their resolution.

**Figure 3:**
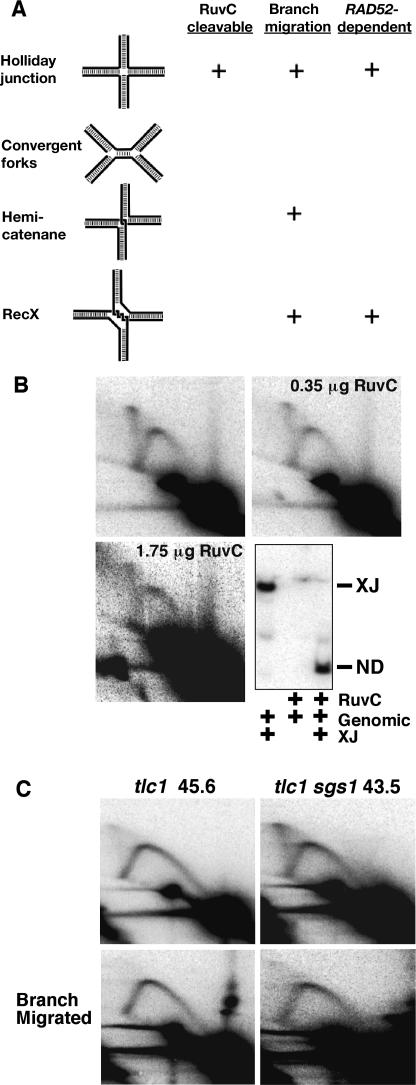
Characterization of Telomere X-Structures (A) Structures of different X-shaped DNA molecules, their susceptibility to cleavage by RuvC, their ability to branch migrate, and their dependence on *RAD52*. (B) ClaI-digested DNA from *tlc1 sgs1* cells at PD 55 was incubated with RuvC buffer alone (top left), with 350 ng (top right) or 1.75 μg (bottom left) of purified RuvC for 3 h at 37 °C prior to 2DGE. Note that the blot of sample treated with the highest level of RuvC is a longer exposure to show the similar degree of loss of X-structures and of Y-type replication structures. RuvC (350 ng) under these conditions fully digested a synthetic X-junction (XJ) that was added to the genomic DNA digests (bottom right). In these controls, genomic DNA was present in all reactions at the same concentration used for 2DGE, and XJ DNA and RuvC were added as indicated; bands corresponding to the XJ and the nicked duplex product (ND) are also indicated. A similar lack of selective X-structure cleavage was observed in *tlc1* mutants, and in samples treated between the first and second dimensions (unpublished data). (C) Prior to the second dimension, first-dimension gel slices containing DNA from *tlc1* and *tlc1 sgs1* mutants at the indicated PD were incubated for 4 h in branch migration buffer at 65 °C (bottom panels) or 4 °C as a control (top panels).

We considered several possible identities for the X-structures, including Holliday junctions (HJs; and structurally similar regressed replication fork or “chicken-foot” structures), convergent replication forks, hemicatenanes (HC), and so-called rec-X structures (Figure 3A). HC have an interlink between single strands from two duplexes, and such a link forms in a *RAD52*-independent fashion between sister chromatids behind advancing replication forks, whereas rec-Xs are thought to form in a *RAD52*-dependent fashion at stalled forks when one nascent strand switches its template transiently for the other nascent strand and then returns to the original template (see also Figure 7A) [27,36–38]. The different types of X-structures can be distinguished based on their susceptibility to cleavage by HJ resolvases such as RuvC, their ability to branch migrate, and their dependence on *RAD52*. To characterize the X-structures, we first tested whether they could be cleaved by the HJ-specific resolvase RuvC [39]. RuvC (350 ng) did not cleave telomere X-structures from *tlc1 sgs1* cells, despite fully cleaving a synthetic X-junction added to the same reactions containing yeast DNA (Figure 3B, top left and right, and bottom right panels, and unpublished data). Five-fold higher levels of RuvC (1.75 μg) partially degraded both the replication arc and X-structures, but had no preferential effect on the X-structures (Figure 3B, bottom left panel). Therefore the X-structures are apparently not HJs. Incubation of gel slices from the first dimension in branch migration buffer at 65 °C prior to running the second dimension caused selective loss of the spike and retention of the replication arc in *tlc1* and *tlc1 sgs1* mutants (Figure 3C). The X-structures therefore cannot be convergent forks, because replication forks do not branch migrate [25], thus leaving HC or rec-Xs as possibilities because they do branch migrate [27,36]. Further, the X-structures branch migrated in the presence of Mg^+2^, consistent with a HC or rec-X, but not HJ, identity (unpublished data) [27].

**Figure 4:**
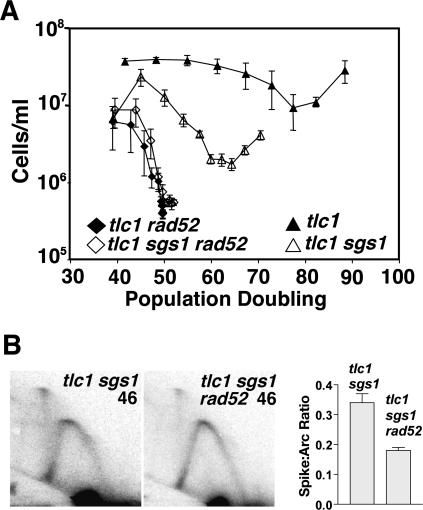
Dependence of Senescence and X-Structures on *RAD52* (A) Effect of *sgs1* and full-ORF *rad52* deletion mutations on the senescence of *tlc1* mutants. Senescence rates were measured in liquid assays for spore products of the indicated genotypes derived from a diploid that was triply heterozygous for *tlc1, sgs1,* and Δ*rad52* mutations. Each value is the mean and standard error of three or four independent spore products. Similar results were obtained using an *est2* deletion allele instead of the *tlc1* mutation (unpublished data). (B) Left and middle: DNA was isolated from *tlc1 sgs1*and *tlc1 sgs1 rad52* mutants at indicated PD and analyzed as in Figure 1B. Right: mean spike:arc ratios for two independent pairs of *tlc1 sgs1* and *tlc1 sgs1* Δ*rad52* cultures were 0.34 ± 0.03 and 0.18 ± 0.01, *p <* 0.04; a second 2DGE of the same two independent pairs of cultures gave similar results. Note that in order to compare PD-matched samples, *tlc1 sgs1* cultures were used prior to the development of high X-structure levels; comparisons made between cultures near the end of senescence show a larger effect of *rad52* deletion (cf. Figure 1B, *tlc1 sgs1* PD60).

To test *RAD52* dependence of the X-structures, we first re-examined the effect of *rad52* mutation on senescence of *tlc1* and *tlc1 sgs1* mutants. This was done because our previous studies used a *rad52* disruption allele beginning after amino acid 167 [22,40], potentially allowing for expression of the N-terminus. Because N-terminal fragments of Rad52p have been shown to be sufficient for DNA binding and strand-annealing activities in vitro [41,42], this raised the possibility that the disruption allele might have residual activity. However, a full-deletion *rad52* allele yielded the same results as the disruption allele: *rad52* deletion sped senescence of *tlc1* mutants, and additional *sgs1* mutation had no further effect on the rate of senescence (Figure 4A), confirming that *RAD52* is epistatic to *SGS1* during senescence. As reported previously [21,43], *RAD52* was required for survivor formation in *tlc1* mutants, because they depend on recombination. Curiously, at later times than those shown, *tlc1 sgs1* Δ*rad52* cells were actually able to form poorly growing survivors, as will be described elsewhere (J. Y. Lee and F. B. Johnson, unpublished data). Comparison on 2DGE of PD-matched cultures of *tlc1 sgs1* with *tlc1 sgs1* Δ*rad52* cells showed a significant 47% reduction, but not elimination of telomere X-structures caused by the *rad52* deletion (Figure 4B and unpublished data). *RAD52* deletion also had no effect on X-structures in *TLC1*+ and early generation *tlc1* mutants prior to significant rise in X-structure levels (Supplementary Figure 2 in Protocol S1, and unpublished data). We conclude that the elevated level, but not the basal level, of X-structures in *tlc1 sgs1* mutants is dependent on *RAD52*. Rec-X formation is *RAD52*-dependent, whereas HC formation is not [27,36], and so, as detailed in the Discussion, a reasonable interpretation of the data is that the basal X-structures are HC and the elevated levels represent rec-Xs formed secondary to stalled replication at telomeres.

**Figure 5:**
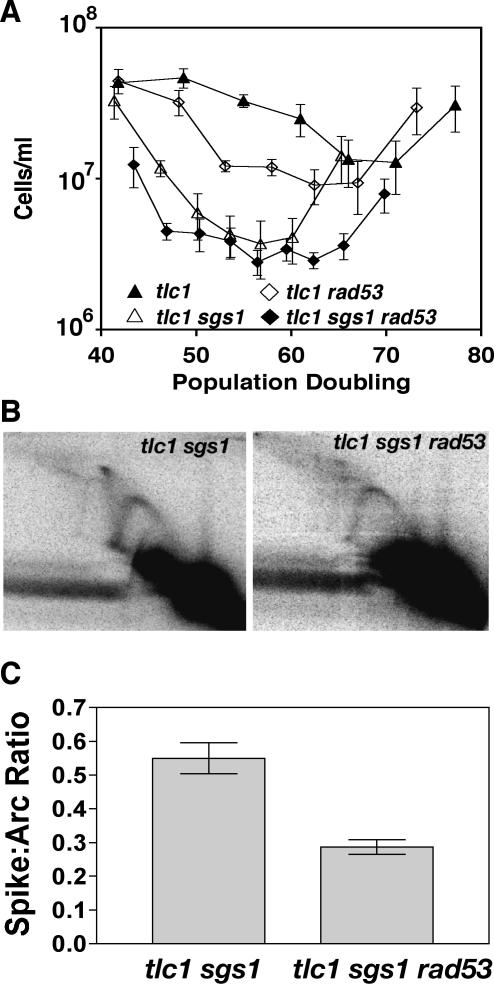
Dependence of Senescence and X-Structures on *RAD53* (A) Effect of *sgs1* deletion and the *rad53K227A* kinase–defective allele on the senescence of *tlc1* mutants. Senescence rates were measured in liquid assays for spore products of the indicated genotypes derived from a diploid that was triply heterozygous for *tlc1, sgs1,* and *rad53K227A* mutations. Each value is the mean and standard error of four independent spore products. (B) DNA was isolated from *tlc1 sgs1* and *tlc1 sgs1 rad53K227A* mutants at PD 55 and analyzed as in Figure 1B. (C) Mean spike:arc ratios for three independent examples each of PD 55 *tlc1 sgs1* and *tlc1 sgs1 rad53K227A* mutants were 0.55 ± 0.05 and 0.29 ± 0.02, respectively, *p <* 0.007)

To further test the idea that the elevated level of X structures might correspond to rec-Xs, we examined the dependence of the telomere X-structures on *RAD53*. At stalled replication forks, Rad53p functions to stabilize the replisome, signal the intra-S phase checkpoint, and facilitate the resumption of replication [6,44]. The kinase-defective *rad53K227A* allele has been shown to lead to the loss of rec-X structures at stalled forks, including suppression of elevated rec-X levels in *sgs1* mutants treated with methane methyl sulfonate [27,36]. *tlc1 rad53K227A* mutants senesced faster than *tlc1* mutants (Figure 5A), consistent with a role for Rad53p in preventing replication fork collapse at telomeres and thus premature senescence in the absence of telomerase. Further, *tlc1 sgs1 rad53K227A* mutants did not senescence significantly faster than *tlc1 sgs1* mutants, as expected if Sgs1p and Rad53p function in the same pathway to prevent rapid senescence. *tlc1 sgs1 rad53K227A* mutants showed a significant 2-fold reduction in X-structure levels compared with PD-matched *tlc1 sgs1* mutants (Figure 5B and 5C), supporting the hypothesis that rec-Xs account for the elevated X-structure levels.

**Figure 6:**
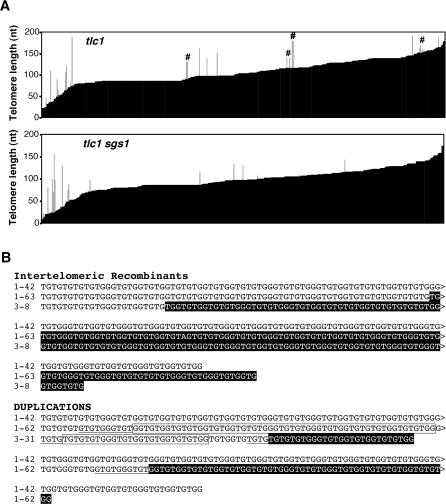
Analysis of Telomere Recombinants by DNA Sequencing (A) Lengths of PCR-amplified, cloned, and sequenced telomere repeat tracts from Chromosome I-L from *tlc1* (top) or *tlc1 sgs1* (bottom) mutant cells are arranged according to the length of nonrecombinant sequences. Nonrecombinant and recombinant tracts are indicated in black or gray, respectively. A total of 434 clones from *tlc1* cells and 439 clones from *tlc1 sgs1* cells were analyzed; the two cell types were sibling spore products and were analyzed at 43.8 and 43.4 PDs after spore germination, well prior to the appearance of survivors. Repeat examples of recombinants that begin at the same position in the nonrecombinant sequence are indicated by a number sign (#). (B) Selected clone sequences from both strains are aligned starting with the first base of the telomere repeats; most of the sequences continue onto a second line (indicated by the greater than symbol [>]). Recombinant sequences are boxed in black. 1–42, 1–62 and 1–63 are *tlc1* clones, and 3–8 and 3–31 are *tlc1 sgs1* clones. Sequence 1–42 is the longest nonrecombinant sequence (178 nt) from the two sets and was used as the reference sequence (the longest *tlc1 sgs1* clone over its entire length matched the 1–42 sequence; unpublished data). Recombinants classified as intertelomeric showed no sequence identity between recombinant tracts and 1–42 sequences, nor with a 263-nt clone derived from earlier generation cells. Recombinants classified as duplications showed identity between the recombinant tract and internal sequences that appear to have been copied (open boxes); underlined sequences indicate the extent of uncertainty of the start of the recombinant tract.

The greater accumulation of X-structures in *tlc1 sgs1* mutants could reflect either increased formation or reduced resolution of recombination intermediates in the absence of Sgs1p. To address these alternatives, and as a second test for telomere recombination during senescence, we used telomere PCR [45] to clone and sequence examples of telomeres from a single chromosome end in the populations of senescing cells. The yeast telomere repeat is imperfect, with an approximate consensus of (TG_1–3_)*_n_,* and individual telomeres thus differ in their precise sequence. Telomerase adds new and variable sequences to telomere ends, but in *tlc1* mutants, the sequence is fixed and typically does not change as a telomere shortens. However, occasional recombination events append new sequences at telomere ends during senescence [46]. If Sgs1p resolves telomere recombination intermediates and thus allows continued cell division, telomere recombinants should be reduced in *tlc1 sgs1* mutants (because these cells would arrest and yield no progeny), whereas if it inhibits the formation of telomere recombination intermediates, an increase in recombinants would be predicted (because recombination events would be more frequent and recombinant cells would yield progeny). Genomic DNA was isolated from *tlc1* and *tlc1 sgs1* mutants at 44 PD after loss of telomerase, far in advance of survivor formation. DNA ends were polyC tailed, and then amplified by PCR using a polyG primer specific to the tail and one specific to sequences internal to the telomere on the left arm of Chromosome I (telomere I-L). Sequence analysis of 434 *tlc1* and 439 *tlc1 sgs1* cloned products showed that, as expected, the majority of the G-rich strands from telomere I-L had a distribution of lengths, which ranged from 8–178 nucleotides (nt), but were identical in sequence over their shared lengths (Figure 6A). The different lengths in the population of senescing cells reflect the stochastic natures of senescence and telomere shortening, as observed previously [46]. Analysis of telomere sequences that diverged from the original showed that 5.1% (22/434) of sequences in *tlc1* mutants were recombinant, similar to the 6.6% reported previously [46]. There was a trend toward fewer recombinants in *tlc1 sgs1* mutants, with recombinants accounting for only 3.9% (17/439) of telomeres (*p* = 0.196). Recombinants included apparent cases of inter-telomeric recombination as well as intra-telomere recombination resulting in duplication of telomere sequences (Figure 6B). Importantly, there was a significant difference in the distribution of recombinants as a function of the length of the nonrecombined telomere repeat tract, with recombinant telomeres occurring at a significantly reduced frequency at longer telomeres in the *tlc1 sgs1* mutants (Figure 6A). Comparing telomeres with nonrecombinant tract lengths of greater than 85 nt, there was a 2.6-fold reduction in the frequency of recombinant telomeres in the double mutants (14/283 vs. 5/264; *p* = 0.026). As detailed in the Discussion, these results can be explained if Sgs1p facilitates the resolution of telomere recombination intermediates and if unresolved intermediates in *tlc1 sgs1* mutants lead to cell cycle arrest, thus inhibiting the accumulation of progeny bearing recombinant telomeres.

**Figure 7:**
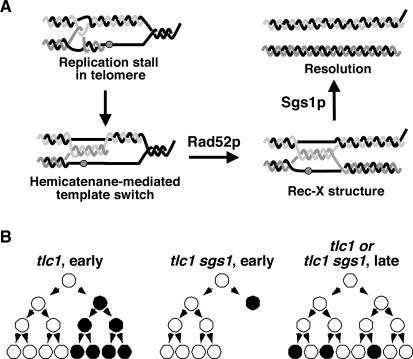
Models for Sgs1p Function during Senescence (A) A stalled replication fork in telomere DNA (barrier represented by an oval) is rescued by HC-mediated template switching, *RAD52*-dependent rec-X formation, completion of DNA replication, and then *SGS1*-dependent resolution. The template switching step is thought to be *RAD52* independent because no strand invasion occurs. Branch migration of rec-X structures in a centromeric direction would generate the molecules observed in the spike region of two-dimensional gels. Sgs1p would cooperate with its Top3p cofactor to enable strand transfers during rec-X resolution; in the absence of Sgs1p, telomeres would remain linked by the HC created by template switching and rec-X formation. (B) Completed telomere recombination (filled circles) early in the lineage of a senescing *tlc1* cell (at a long telomere) gives rise to progeny bearing the recombinant telomere (left). Stalled recombination in a *tlc1 sgs1* cell leading to arrest would not generate progeny (middle). Whether completed or not, recombination at the end of senescence (at a short telomere) does not give rise to progeny (right).

## Discussion

Previous studies have indicated that RecQ helicases, including the human WRN and BLM and S. cerevisiae Sgs1p, are important in the maintenance of telomeres. Here, we have used physical and genetic methods to investigate how Sgs1p slows the rate of senescence in yeast *tlc1* mutants. Our new findings indicate a role for Sgs1p in the resolution of telomere recombination intermediates and lend mechanistic insight to earlier observations of the function of Sgs1p during senescence [15,17,22]. Further, they may help explain telomere defects caused by deficiencies in other RecQ helicases.

We observed an accumulation of X-shaped structures at telomeres in *tlc1 sgs1* mutants. Because RuvC cannot selectively cleave these X-structures, they do not appear to be HJs. This is supported by our recent report that the C-terminal 200 amino acids of Sgs1p are dispensable for slowing senescence [22]; this C-terminus contains the HRDC domain, which is important for HJ binding and double-HJ dissolution [47–49], arguing that HJ-targeted functions of Sgs1p are not involved in slowing senescence. These X-structures can branch migrate, indicating that they cannot be convergent replication forks, but this is consistent with them being HC or rec-X structures. In *S. cerevisiae,* HC have been observed to form behind replication forks in a *RAD52*-independent fashion [36,38]. Liberi et al. [27] suggested that at a stalled fork, resumption of replication might use a HC to facilitate template switching whereby one nascent strand leaves the parental template and copies the other nascent strand (to bypass the cause of the stall) before returning to the original template in a *RAD52*-dependent step, thus forming a rec-X structure. Our finding that the elevated level, but not the basal level, of X-structures in *tlc1 sgs1* mutants is *RAD52-* and *RAD53*-dependent is consistent with the elevated level structures being rec-Xs and the basal level structures being HC. According to this model (Figure 7A), the stalling of replication forks that occurs naturally in the telomere repeats [24,50] might be somehow enhanced by changes related to telomere shortening (see below) and thus might lead to rec-X formation and eventual resolution by Sgs1p; in the absence of Sgs1p, rec-X structures would accumulate. This is analogous to the recently reported accumulation of rec-X structures at non-telomeric loci in *sgs1* mutants after the stalling of replication forks by methyl methane sulfonate [27]. Sgs1p would be expected to function in tandem with its Top3p cofactor to effect strand transfer reactions that would enable resolution of the rec-X structure [27,51,52], consistent with our finding that such cooperation is required to prevent rapid senescence [22]. If unresolved, rec-Xs might lead directly to cell cycle arrest; if resolved by other means (e.g., nucleases), the shortened or aberrantly structured telomere ends might hasten the onset of senescence. In *TLC1*+ cells, telomerase could repair such ends, thus explaining the normal telomere length in *sgs1* mutants and the synergy of *sgs1* mutation with *tlc1* mutation to accelerate senescence. Previously, we observed that senescent *tlc1 sgs1* mutants appear unable to segregate nuclei between mother and daughter cells [15], and a possible explanation is that unresolved recombination intermediates interfere with chromosome segregation. We note also that suppression by Sgs1p of X-structure accumulation at stalled replication forks was recently shown to cooperate with a parallel pathway that is dependent on SUMOylation [18]. This might explain our recent observation that, like Sgs1p, Slx5p and Slx8p are required to prevent rapid senescence of *tlc1* mutants, because Slx5p and Slx8p function in parallel with Sgs1p for cell viability and also show genetic interaction with SUMO pathway factors [22,53].

It is not yet clear why telomere shortening should increase X-structure levels at telomeres, although one possibility is that changes in chromatin might contribute to increased replication fork stalling and thus rec-X formation. For example, decreased Rap1p binding at shortened telomeres might allow telomere repeats to adopt DNA structures involving hydrogen bonding between guanines, e.g., G-quadruplexes, that could impede replication. The recent demonstration of impaired telomere replication in S. pombe cells lacking the Taz1 telomere repeat binding protein supports this idea, and led the authors to also propose that Rap1p might serve a similar function to facilitate telomere replication in *S. cerevisiae* [54]. Of note, however, a mutant Rap1p protein lacking the C-terminus does not impact telomere fork stalling [24], although this mutant possesses the N-terminal DNA binding domain and so might have retained the function proposed to facilitate replication. We further note that we did not observe in senescing *tlc1* or *tlc1 sgs1* cells an increase in apparent stalling near the 2N spot, corresponding to the telomere repeats, although this might be explained by efficient conversion of stalled forks into rec-X structures. A second possibility is that a shortened and less heterochromatic telomere might be more accessible to recombination factors like Rad52p, thus facilitating rec-X formation. The elevated recombination rates at shortened telomeres in K. lactis and telomerase knockout mice are consistent with these possibilities [55,56].

During senescence, Sgs1p may inhibit the formation of telomere recombination intermediates, or facilitate their resolution. Using telomere PCR and sequencing, we observed a significantly decreased frequency of recombinants occurring at longer telomeres in *tlc1 sgs1* mutants, supporting the model that Sgs1p helps resolve telomere recombination intermediates into mature products. For this reason, we suggest that decreased resolution, rather than increased formation of X-structures explains their higher levels in *tlc1 sgs1* mutants. Two aspects of the PCR assay used to detect the recombinant telomeres must be understood to explain why recombinants are less frequent at longer telomeres in the absence of Sgs1p. First, the number of cell divisions after loss of telomerase at which a recombinant arises will influence the apparent frequency of that event: productive recombination events that occur early and give rise to progeny will remain at a frequency approximately equal to that at the time of their occurrence, whereas events that occur late will appear at a lower frequency that reflects the larger size of the pool of cells at that later time point (Figure 7B, left vs. right). Although only one time point was examined for each strain, the stochastic natures of senescence and telomere shortening caused some cells to be closer to senescence than others, and so information spanning a large range of telomere lengths was obtained. The second aspect of the telomere PCR assay that must be appreciated is that it will detect both resolved products and unresolved intermediates, and therefore, any stalled recombination intermediates in cells lacking Sgs1p will still be observed; this fact minimizes the measured difference in recombinants between *tlc1* and *tlc1 sgs1* mutants when telomeres of all lengths are examined (because recombinants forming at short telomeres are expected to suffer little from the *sgs1* defect; see below). Telomere recombination appears to increase as telomeres shorten (Figure 1D and [28]), yet the frequency of recombinants measured by telomere PCR in *tlc1* cells was not greater at shorter than at longer telomeres. A reasonable explanation is that the recombination events at short telomeres were more likely to have occurred in cells that were closer to senescence and so gave rise to fewer progeny than the cells experiencing recombination at long telomeres. These competing effects of more frequent recombination at short telomeres but fewer recombinant progeny arising from cells with short telomeres could balance each other in the *tlc1* mutants so that the distribution of recombinants is similar among telomeres of all sizes. In contrast, if Sgs1p is required for efficient resolution of recombination intermediates and if unresolved intermediates cause cell cycle arrest, then cells with long telomeres, and thus high replicative potential, would be most affected by stalled recombination events; *tlc1 sgs1* mutants arrested by stalled recombination intermediates at long telomeres will become diluted by the other dividing cells (Figure 7B, middle). Cells with short telomeres are unlikely to divide much further regardless of the outcome of a telomere recombination event, and thus absence of Sgs1p would have relatively little effect on the measured frequency of recombinants at short telomeres (Figure 7B, right). This explains why the decrease in recombinants in the *tlc1 sgs1* mutants occurs preferentially at longer telomeres. As an interesting aside, by this view, the distribution of recombinants among telomeres of different lengths in *tlc1 sgs1* mutants most accurately reflects the propensity of short telomeres to engage in recombination because this distribution is not skewed, as it is in *tlc1* mutants, by the opposing effect of recombination at longer telomeres tending to occur in cells with greater remaining replicative potential and thus giving rise to more progeny. Consistent with the interpretation that stalled recombination events in *tlc1 sgs1* mutants lead to permanent cell cycle arrest, WRN is required in cultured human cells for the resolution of recombination intermediates that enable cells to generate viable progeny [57].

If *tlc1* mutants can complete telomere recombination and give rise to viable progeny, then repeat examples of the same recombination event should be detectable. Indeed, four independent examples were obtained in the *tlc1* cells (indicated by the number sign [#], Figure 6A). No such repeat events were observed in the *tlc1 sgs1* cells, consistent with telomere recombination often being a terminal event in the absence of Sgs1p. Furthermore, no such events were observed in the shortest (<85 nt) telomeres of *tlc1* mutants, consistent with the recombination events at short telomeres occurring in cells that are near the end of their lifespan.

The action of Sgs1p during senescence need not reflect any telomere-specific function, but rather may be one manifestation of a general role in the restart of replication forks stalled for various reasons, for example, hydroxyurea treatment, DNA alkylation by methane methyl sulfonate, or as proposed here, chromatin changes at shortened telomeres. We note, however, that stalled forks in the terminal telomere repeats would be particularly problematic because there is no replication origin distal to the stall to generate a rescuing fork, thus perhaps contributing to the dependence of telomeres on Sgs1p-dependent restart during senescence. Sgs1p helps activate the checkpoint response to DNA damage in S phase, and also helps to stabilize DNA polymerases alpha and epsilon at stalled forks [35,58]. The former function, but not the latter, is thought to occur in collaboration with Rad53p [58]. Nonetheless, Rad53p appears to help stabilize stalled replication forks [27,36,59,60], although the extent to which this reflects stabilization of DNA polymerases [35,61], the MCM helicase [62], or other functions, and the degree to which Sgs1p is required for these functions, are not resolved at present. The reduction in X-structure levels caused by the *rad53K227A* allele and modest acceleration of senescence in *tlc1 rad53K227A* mutants is consistent with the model that rec-X–dependent fork restart contributes to optimal telomere replication during senescence (Figure 7A). The larger effect of *sgs1* mutation on senescence may reflect a hypomorphic effect of the *rad53K277A* allele with respect to HC- and rec-X–mediated fork rescue, such that some stalled forks are still routed through this pathway and thus depend on Sgs1p function. Alternatively, the capacity of Sgs1p to stabilize stalled forks may be greater than that of Rad53p. Our findings leave open the possibility that replisome stabilization by Sgs1p may contribute to slowing senescence, in addition to the proposed role in rec-X resolution.

Given the increased loss of telomeres replicated by lagging-strand synthesis in WS cells [11], it is interesting that fork stalling was not increased in *sgs1* mutants. Therefore, Sgs1p is not required for telomere replication in most instances. However, the WS defect affects only about 2% of telomeres [11], and it is possible that similarly infrequent replication defects that are below the limit of detection of the 2DGE assay do occur in *sgs1* mutants. Another possibility is that WRN has a function in telomere replication that is different from Sgs1p. However, we note that the helicase domain of WRN, which is conserved among all RecQ family helicases, is critical for its telomere maintenance function [11], and further, that human and mouse BLM [10,13] and a S. pombe RecQ homolog [7] also appear to have roles in telomere maintenance, and so this likely represents a conserved function of several RecQ proteins. Perhaps recombination defects like those observed here in *tlc1 sgs1* mutants contribute to the replication-related telomere defects of WS cells. If so, our model does not address why the defect in Werner cells should selectively affect the telomere strand copied by lagging-strand synthesis. Given the propensity of RecQ proteins to unwind G-quadruplexes [48], one possible explanation is that, in the absence of a RecQ helicase, persistence of a G-quadruplex on the unpaired G-rich strand of a rec-X intermediate might lead to cleavage by a G-quadruplex–specific nuclease (e.g., Mre11 [63]) and thus, selective loss of this strand. Alternatively, differences in the structure of the termini at telomeres generated by lagging- versus leading-strand synthesis may affect the propensity for recombination, since the product of lagging-strand synthesis has a 3′ overhang, whereas the initial leading strand product would have a less recombinogenic blunt end. Further investigation of these possibilities, and of the interface between replication, recombination, and telomere maintenance, should improve understanding of the mechanisms underlying the cancer and age-related diseases caused by deficiencies in RecQ helicases.

## Materials and Methods

### Yeast strains and senescence assays.

All strains were isogenic derivatives of YBJ133 *(Mata/*α Δ*ho* Δ*hml::ADE1* Δ*hmr::ADE1 ade1 ura3–52, leu2–3,112, lys5, TLC1/*Δ*tlc1::kanMX,* and *SGS1/*Δ*sgs1*::*hisG*-*URA3)* [15]. *RAD52* was deleted by PCR-mediated open reading frame (ORF) replacement with *LEU2* in one allele of YBJ133 to generate YJL4. The *rad53K227A* allele was introduced using plasmid pCH8 as described [64]. into a YBJ133 derivative that was *TLC1/*Δ*tlc1::HygB,* rather than *TLC1/*Δ*tlc1::kanMX,* to generate YBJ436. Senescence experiments were as described [15], using haploid spore products derived from diploids that were heterozygous for mutations in *TLC1, SGS1,* and in some cases, *RAD52 or RAD53,* and cells were cultured at 30 °C in standard YPD medium.

### Genomic DNA isolation, electrophoresis, and Southern analysis.

DNA was purified from log-phase cells using hexamine cobalt (III) to limit branch migration as described [65]. Telomere length was measured by Southern analysis of XhoI-digested DNA using a Y′ probe as described [15]. 2DGE for telomere analysis was as described using ClaI-digested DNA [50], except the first dimension was run at 4 °C at 0.6 V/cm for 68–70 h, and the second dimension was at 3 V/cm for 20–22 h. The Y′L-specific probes were generated by amplification of genomic DNA with primers 5′-ggcgttgcaatgtggaaatg and 5′-gaccggcaaaagcgagtagc. The 2DGE for rDNA analysis was performed as for telomeres except that DNA was digested with SnaBI, and the first dimension was for 19 h at 1 V/cm, and the second dimension was for 4 h at 4.8 V/cm. The rDNA probe (to the 18S ribosomal RNA region) was generated using PCR amplification of genomic DNA with primers 5′-CTGGTGAGTTTCCCCGTGTTGAG and 5′-CCTTGTGCTGGCGATGGTTC. ^32^P-probed blots were washed and visualized as described [15] using a Molecular Dynamics Phosphoimager (Molecular Dynamics/GE Healthcare, http://www.gehealthcare.com/usen/index.html), and levels of X-structures, replication forks, and 1N and 2N spots were determined with ImageQuant software. For branch migration, the first-dimension gel slice was incubated in 10 mM Tris-HCl (pH 8.0), 0.1 mM EDTA, and 100 mM NaCl (and in additional tests, with 10 mM MgCl_2_) at 4 °C for 2 h and then at 65 °C for 4 h. For RuvC treatment, ClaI-digested DNA was ethanol precipitated and dissolved in 45 ml of RuvC buffer (12 mM Tris-Cl [pH 8.0], 10 mM MgCl_2_, 1 mM DTT, and 100 μg/ml BSA) and treated with 350 ng of one of two different preparations of purified RuvC (Gifts of the M. Whitby and R. Lloyd laboratories; similar results were obtained with both preparations) at 37 °C for 3 h. To confirm RuvC activity, the synthetic X-junction J11 was constructed as described [66], 5 ng was added to control digests that were otherwise identical to experimental digests, reactions were separated on 10% native PAGE gels, and the blotted products probed with ^32^P end-labeled oligonucleotide J11–2 [66].

### Telomere PCR.

Telomere PCR was performed as described [45] with modifications. DNA was isolated using a Qiagen genomic DNA kit (http://www1.qiagen.com), denatured, C-tailed, and the telomere ends of Chromosome I-L were amplified using o286S MluI: 5′-CACGCGTGGTTGGCCAGGGTTAGATTAGGGCTG-3′ and an equimolar mixture of G18A BamHI: 5′-CGGGATCCG_18_A-3′ and G18C BamHI: 5′-CGGGATCCG_18_C-3′. Unexpectedly, one of the Chromosome I copies in YBJ133 possessed an uncharacterized polymorphism that prevented amplification; this was discovered because there was a 2:2 segregation in the ability of DNA from spore products to be amplified; conveniently, this ensured that all amplifiable spore products inherited the same telomere. PCR amplification included 1.25 M betaine because its addition to control reactions with a 262-base pair (bp) cloned telomere repeat sequence yielded a sharp band of the expected size that was more distinct and at higher yield than standard conditions. PCR products were separated on 2% agarose gel, 50–1,000-bp fragments were excised and cloned into the pCR4-TOPO vector (Invitrogen, http://www.invitrogen.com), transformed into DH5α Escherichia coli cells, and individual clones were sequenced by Certigen (http://www.certigen.com) or by the University of Pennsylvania DNA Sequencing Facility. Telomere sequences were aligned using MegAlign software and compared using the National Center for Biotechnology Information BLAST. Rules used to classify telomere sequences are detailed in Supplementary Figure 3 in Protocol S1.

### Statistics.

Two-tailed unpaired *t*-tests were performed for all comparisons except the frequency of recombinants, for which a one-tailed chi-square test was performed.

## Supporting Information

Protocol S1 Supplementary References and Figures 1, 2, and 3
(603 KB PDF)

## Abbreviations

2DGE: two-dimensional gel electrophoresis
ALT: alternative lengthening of telomeres
BLM: Bloom syndrome protein
HC: hemicatenane
HJ: Holliday junction
PD: population doubling
WRN: Werner syndrome protein
WS: Werner syndrome
Y′L: Y′ long
Y′S: Y′ short
