# Supplementary material for: Evidence That a RecQ Helicase Slows Senescence by Resolving Recombining Telomeres
Source: PLoS Biol. 2007 Jun 5;5(6):e160. doi: 10.1371/journal.pbio.0050160 (PMC1885831; doi:10.1371/journal.pbio.0050160)
Supplement: Supplementary file 1 [file pbio.0050160.sd001.pdf]

Supplementary Figure 1. Telomere replication and X structures in senescing *tlc1* and *sgs1 tlc1* mutants visualized with a probe that recognizes both Y'S and Y'L elements. (A) Two-dimensional gel electrophoresis (2DGE) separates DNA by size then shape so that Y-shaped replication structures appear along the replication arch (RA) (1). X-structures (XS) appear as a spike extending from the 2N spot. **(B)** Genomic DNA was harvested from WT, *sgs1, tlc1*, and *tlc1 sgs1* cells in log-phase at the population doubling indicated (*upper right of each panel*), digested with ClaI, separated by 2D electrophoresis, blotted and probed for Y' DNA with the 784 bp probe just subtelomeric to the $TG_{1\text{-}3}$ repeats as described (2).The most prominent replication arch and spike *(indicated by arrow*) are from terminal Y'L DNA (◆), the largest arch is from internal Y'L DNA (●), and the smallest arch and spike are from internal Y'S DNA (■); these assignments are the same as those made previously in a similar study (3). Note the increased spike levels in *tlc1* and *tlc1 sgs1* mutants.

A

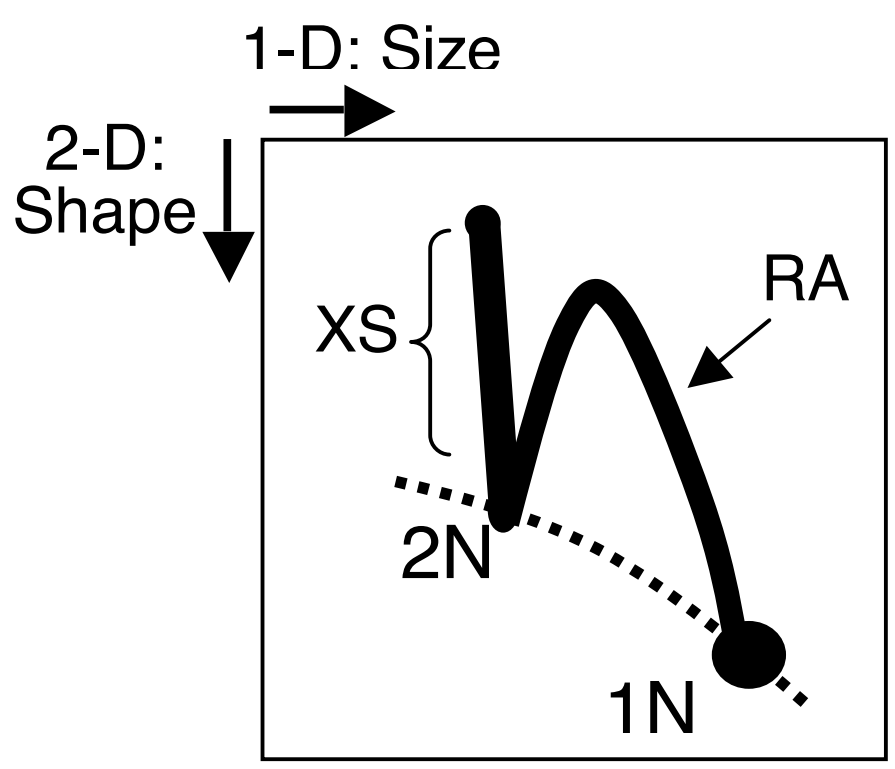

B

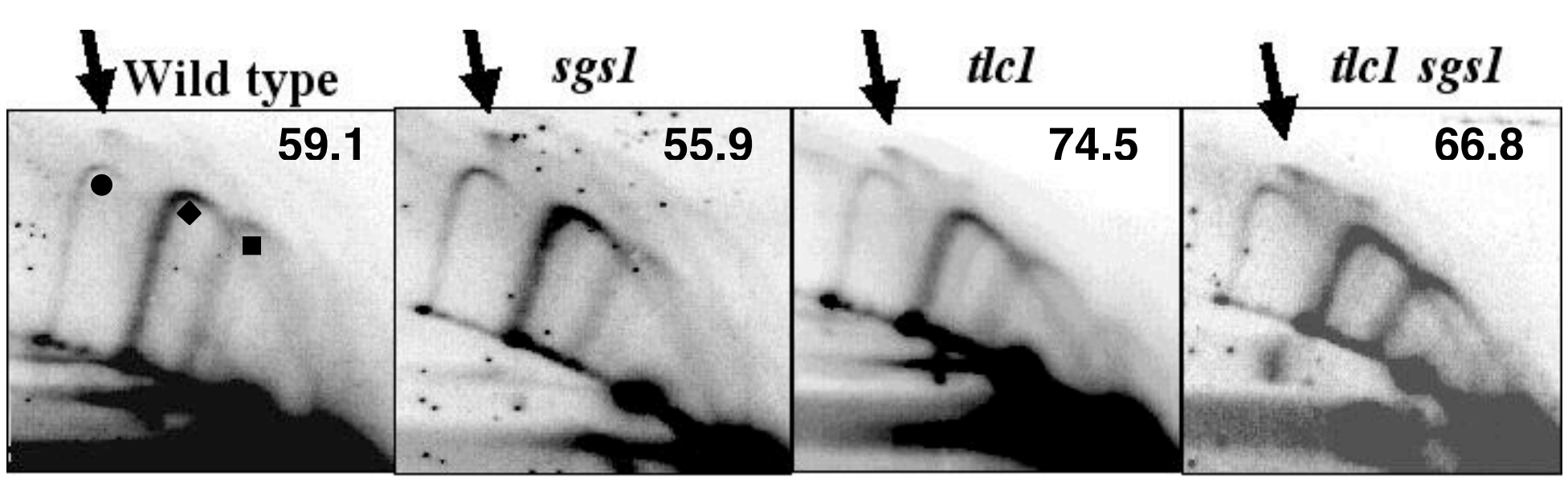

Supplementary Figure 2. ***RAD52*-independence of basal X-structures.** DNA was isolated from *tlc1* and *tlc1 rad52* mutants at indicated population doublings and analyzed by 2DGE and Southern analysis as in Figure 1. No significant reduction in telomere X-structure level was caused by *rad52* deletion at this early PD, and there was also no effect on telomere X-structure levels in wild type or *sgs1* mutants (data not shown).

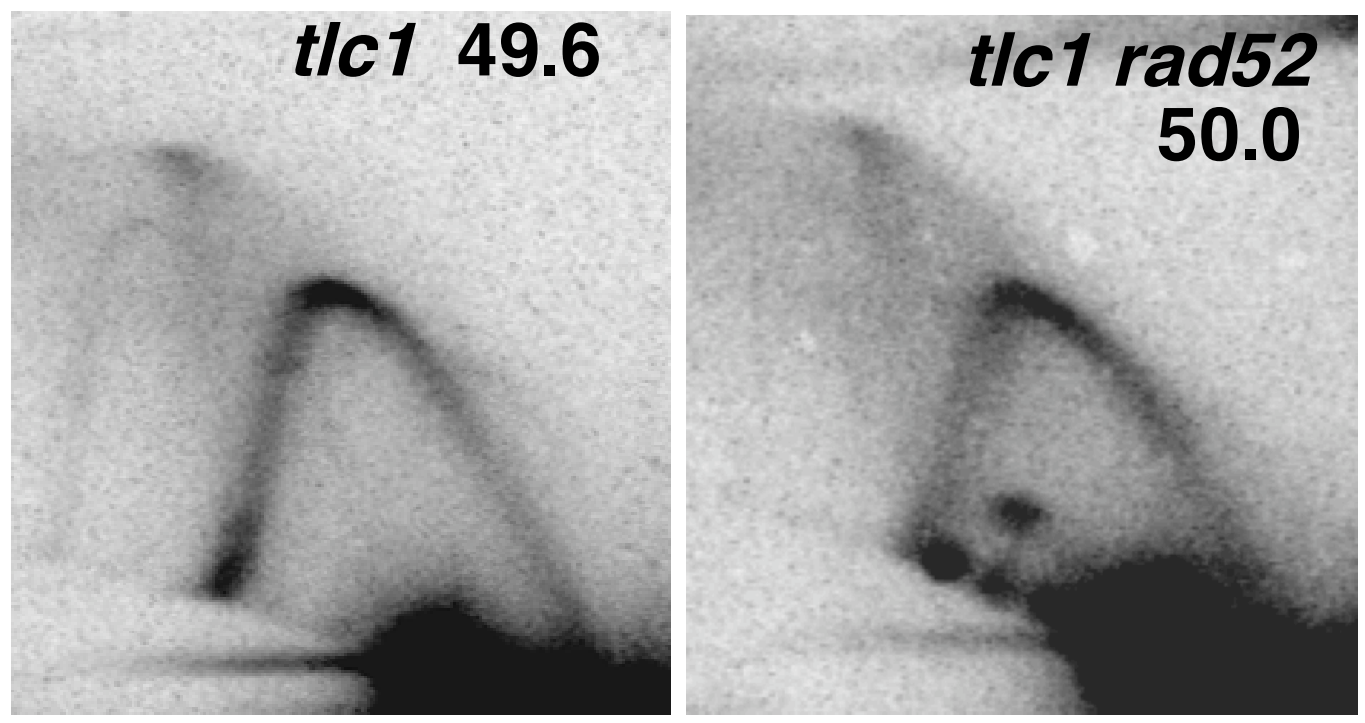

Supplementary Figure 3. Sequences of all recombinant telomeres.

Because of the partially repeated structure of the yeast telomere, the precise nature of every sequence alteration cannot be unambiguously defined, and thus sequence analysis does not provide information on absolute telomere recombination frequencies. We used the following system to classify telomere sequences, which allowed us to objectively compare *differences* in recombinant frequencies between *tlc1* and *tlc1 sgs1* mutants:

Non-recombinant sequences included 1) perfect matches to the consensus, 2) single point mutations, 3) single deletions or insertions of 1 or 2 nucleotides that realigned the sequence with the consensus.

Recombinants included 1) intertelomeric recombinants, defined as sequence alterations that could not be aligned with any part of the consensus sequence, and 2) duplication recombinants, defined as sequence alterations that could be aligned with a segment (or segments) of the consensus centromeric to the breakpoint, and 3) multiple nucleotide insertion/deletion recombinants, defined as alterations that might be accounted for by simple insertions or deletions but that required insertion or deletion of more than two nucleotides to confer alignment (and so can as easily be explained as intertelomeric recombinants).

Duplicated telomere sequences in recombinants could be generated by slippage during template switching, and intertelomeric recombination events might be generated if a switching strand invades a different telomere (or extrachromosomal telomere-repeat-containing molecule); the clustering of telomeres in the yeast nucleus might facilitate such events (4).

The true frequency of template switching events cannot be determined by the telomere PCR assay because most may involve faithful copying of nascent sister strands and so would not cause a change in telomere sequence. Further, the intertelomeric class may be underestimated because it is possible, especially in cases with short recombinant tracts, that the copied sequences exist at telomeres in addition to the one examined (I-L). Thus the origin of the copied sequences could be from telomere I-L, another telomere, or extrachromosomal DNA containing telomere repeats.

Legend:

1-42: Reference sequence used in sequence alignment and "BLAST 2 Sequences"

Highlighted sequences: New sequences added to the telomere end due to a recombination event.

Boxed sequences: Sequences in the core consensus that align with the new recombinant sequences suggesting the occurrence of a duplication event. In cases where more than one source of the duplicated sequence is possible, only the first such sequence centromeric to the breakpoint is shown.

Underlined sequences: Upstream sequences of the new recombinant sequence that align with sequences upstream of the duplicated sequence.

Highlighted sequences: Inserted sequences

---: Deleted sequences

Bold: Single nucleotide change

Sequences:

1) *tlc1* Recombinant Sequences

Intertelomeric Recombinants:

```
1-284 TGTGTGTGTGTGGGTGTGGTGTGGTGTGTGGTGTGGTGTGTGGGTGTGGGTGCGGGTGTGGTGTGGGTGTG
1-34  TGTGTGTGTGTGGGTGTGGTGTGGTGTGTGGTGTGTGGTGTGGTGTGTGGTGTGGGTGTGTGTGGGTGTGG
1-63  TGTGTGTGTGTGGGTGTGGTGTGGTGTGTGGTGTGGTGTGTGGGTGTGTGGGTGTGGTGTGTGTGGTGTGT
1-79  TGTGTGTGTGTGGGTGTGGTGTGGTGTGTGGTGTGGTGTGTGGGTGTGTGGGTGTGGTGTGTGTGGTGTGT
1-42  TGTGTGTGTGTGGGTGTGGTGTGGTGTGTGGTGTGGTGTGTGGGTGTGTGGGTGTGGTGTGTGTGGTGTGT

1-284 TGGGTGTGGTGTGTGGG
1-34  TGTGTGGGTGTGGGTGTGGTGTGGGTGTGGTGTGTGGG
```

```
1-63  GTGTGTGGGTGTGGTGTGGTGTGTGGTGTAGTGTGTGGGTGTGTGGGTGTGGTGTGTGGTGTGTGGGTGTG
1-79  GGGTGTGGGTGTGGTGTGGGTGTGGGTGTGGTGTGTGGGTGTGGGTGTGGTGTGGGTGTGGGTGTGGTGTG
1-42  GGGTGTGGGTGTGGTGTGGGTGTGGGTGTGGTGTGTGGGTGTGGGTGTGGTGTGGGTGTGGGTGTGGTGTG

1-63  GGTGTGGTGTGGGTGTGGGTGTGTGTGTGGGTGTGGGTGTGGTG
1-79  TGTGGGTGTGGGTGTGGGTGTGGGTGTGTGTGGGTGTGTGTGGGTGTG
1-42  TGGGTGTGGTGTGGGTGTGGTGTGGGTGTGGTGTGG
```

Duplications:

```
1-74  TGTGTGTGTGTGGGTGTGGTGTGGTGTGTGGTGTGGTGTGTGGGTGTGTGGGTGTGGTGTGTGTGGTGTGT
1-39  TGTGTGTGTGTGGGTGTGGTGTGGTGTGTGGTGTGGTGTGTGGGTGTGTGGGTGTGGTGTGTGTGGTGTGT
1-280 TGTGTGTGTGTGGGTGTGGTGTGGTGTGTGGTGTGGTGTGTGGGTGTGTGGGTGTGGTGTGTGTGGTGTGT
1-62  TGTGTGTGTGTGGGTGTGGTGTGGTGTGTGGTGTGGTGTGTGGGTGTGTGGGTGTGGTGTGTGTGGTGTGT
1-50  TGTGTGTGTGTGGGTGTGGTGTGATGTGTGGTGTGGTGTGTGGGTGTGTGGGTGTGGTGTGTGTGGTGTGT
1-57  TGTGTGTGTGTGGGTGTGGTGTGGTGTGTGGTGTGGTGTGTGGGTGTGTGGGTGTGGTGGGTGTGGTGTGT
1-253 TGTGTGTGTGTGGGTGTGGTGTGGTGTGTGGTGTGGTGTGTGGGTGTGTGGGTGTGGTGTGTGTGGTGTGT
1-42  TGTGTGTGTGTGGGTGTGGTGTGGTGTGTGGTGTGGTGTGTGGGTGTGTGGGTGTGGTGTGTGTGGTGTGT

1-74  GGGTGTGGGTGTGGTGTGTGTGTGGGTGTGGTGTGTGTGGTGTGTGGGTGTGGGTGTGG
1-39  GGGTGTGGGTGTGGTGTGGGTGTGGGTGTGGTGTGTGGGTGTGGTGTGGGTGTGGGTGTGGTGCGTGG
1-280 GGGTGTGGGTGTGGTGTGGGTGTGGTGTGGTGTGTGGGTGTGTGGGTGTGGTGTGTGTGGTGTGTGGG
1-62  GGGTGTGGGTGTGGTGTGGGTGTGGTGTGGTGTGTGGTGTGGTGTGTGGGTGTGTGGGTGTGGTGTGTGTG
1-50  GGGTGTGGGTGTGGTGTGGGTGTGGTGTGGTGTGTGGTGTGGTGTGTGGGTGTGTGGGTGTGGTGTGTGTG
1-57  GGGTGTGGGTGTGGTGTGGGTGTGGGTGTGGTGTGTGGGTGTGGGTGTGGTATGGGTGTGGGTGTGGGTGT
1-253 GGGTGTGGGTGTGGTGTGGGTGTGGGTGTGGTGTGTGGGTGTGGTGTGTGTGGTGTGTGGGTGTGGGTGTG
1-42  GGGTGTGGGTGTGGTGTGGGTGTGGGTGTGGTGTGTGGGTGTGGGTGTGGTGTGGGTGTGGGTGTGGTGTG

1-62  GTGTGTGG
1-50  GTGTGTGGGTGTGGGTGTGG
1-57  GGTGTGGGTGTGGGTGTGGTG
1-253 GTGTGGGTGTGGGTGTGGTGTG
1-42  TGGGTGTGGTGTGGGTGTGGTGTGGGTGTGGTGTGG
```

Multiple Nucleotide Deletions & Insertions:

```
1-271 TGTGTGTGTGTGGGTGTGGTGTG-----TGGTGTGGTGTGTGGGTGTGTGG
1-13  TGTGTGTGTGTGGGTGTGGTGTGGTGTGTGGTGTGGTGTGTGGGTGTGGGTGTGGGTGTGGTGTG
1-5   TGTGTGTGTGTGGGTGTGGTGTGGTGTGTGGTGTGGTGTGTGGGTGTGTGGGTGTGGTGTGTGTGGTGTG-
1-35  TGTGTGTGTGTGGGTGTGGTGTGGTGTGTGGTGTGGTGTGTGGATGTGTGGGTGTGGTGTGTGGGTGTGTG
1-148 --TGTGTGTGTGGGTGTGGTGTGGTGTGTGGTGTGGTGTGTGGGTGTGTGGGTGTGGTGTGTGTGGTGTGT
1-49  TGTGTGTGTGTGGGTGTGGTGTGGTGTGTGGTGTGGTGTGTGGGTGTGTGGGTGTGGTGTGTGTGGTGTGT
1-152 TGTGTGTGTGTGGGTGTGGTGTGGTGTGTGGTGTGGTGTGTGGGTGTGTGGGTGTGGTGTGTGTGGTGTGT
1-160 TGTGTGTGTGTGGGTGTGGTGTGGTGTGTGGTGTGGTGTGTGGGTGTGTGGGTGTGGTGTGTGTGGTGTGT
1-42  TGTGTGTGTGTGGGTGTGGTGTGGTGTGTGGTGTGGTGTGTGGGTGTGTGGGTGTGGTGTGTGTGGTGTGT

1-5   -------GGTGTGGTGTG
1-35  GGTGTGGTGTGTGTGGTGTGTGGGTGTGGGTGTGG
1-148 GGGTGTGGGTGTGGTGT-----------------GTGGGTGTGGGTGTGGTGTGGGTGTGGGTGTGG
1-49  GGGTGTGGGTGTGGTGTGGGTGTGGGTGTGGTGTGTGGGTGTGGGTGTGGTGTGGGTGTGGGTGTGGTGTG
1-152 GGGTGTGGGTGTGGTGTGGGTGTGGGTGTGGTGTGTGGGTGTGGGTGTGGTGTGGGTGTGGGTGTGGTGTG
1-160 GGGTGTGGGTGTGGTGTGGGTGTGGGTGTGGTGTGTGGGTGTGGGTGTGGTGTGGGTGTGGGTGTGGTGTG
1-42  GGGTGTGGGTGTGGTGTGGGTGTGGGTGTGGTGTGTGGGTGTGGGTGTGGTGTGGGTGTGGGTGTGGTGTG
```

```
1-49  TGGGTGTGG-----GTGTGGTG
1-152 TGGGTGTGG-----GTGTGGTGTGGG
1-160 TGGGTGTGG-----GTGTGGTGTGGGTGTGG
1-42  TGGGTGTGGTGTGGGTGTGGTGTGGGTGTGGTGTGG
```

NOTES:
1-74, 1-39, 1-253 are recombinant sequences each with an identical replica representing repeat examples of the same recombination event
1-49. 1-152. 1-160 are recombinant sequences that have the same breakpoint but different lengths of distal sequence, and so may represent three progeny of one recombination event with different extents of telomere shortening among the progeny.

2) *tlc1 sgs1* Recombinants

Intertelomeric Recombinants:

```
3-315 TGTGTGTGGGTGGTGTGTGTG
3-80  TGTGTGTGTGTGGGTGTGGTGTGTGTGGTGTGTGGGTGTGGGTGTGGTGTGTGTGGTGTGTGGGTGTGG
3-260 TGTGTGTGTGTGGGTGTGGGTGTGGTGTGTGTGGTGTGTGGGTGTGGGTGTGGTGTGTGTGGTGTGTGGTG
3-115 TGTGTGTGTGTGGGTGTGGTGTGGTGTGTGGTGTGGTGTGTGGGTGTGGGTGTGGGTGTGGTGTGGGTGTG
3-34  TGTGTGTGTGTGGGTGTGGTGTGTGGGTGTGGTGTGTGGGTGTGGGTGTGGTGTGTGGGTGTGGGTGTGGG
3-65  TGTGTGTGTGTGGGTGTGGTGTGGTGTGTGGTGTGGTGTGTGGGTGTGTGGGTGTGGTGTGTGTGGTGTGT
3-8   TGTGTGTGTGTGGGTGTGGTGTGTGGTGTGGTGTGTGGGTGTGTGGGTGTGGTGTGTGTGGTGTGTGTGTG
1-42  TGTGTGTGTGTGGGTGTGGTGTGGTGTGTGGTGTGGTGTGTGGGTGTGTGGGTGTGGTGTGTGTGGTGTGT

3-260 TGGGTGTGG
3-155 TGGGTGTGGTGTGTGG
3-34  TGTGGTGTGGGTGTGTGGGTGTGGTGTG
3-65  GGGTGTGGGTGTGGGTGTGGGTGTGGGTGTGGGTGTGGTGTGG
3-8   TGGGTGTGGTGTGTGTGTGGGTGTGGTGTGTGGGTGTGGGTGTGGTGTGGGTGTGGGTGTGGTGTGTGGGT
1-42  GGGTGTGGGTGTGGTGTGGGTGTGGGTGTGGTGTGTGGGTGTGGGTGTGGTGTGGGTGTGGGTGTGGTGTG

3-8   GTGGGTGTGGTGTG
1-42  TGGGTGTGGTGTGGGTGTGGTGTGGGTGTGGTGTGG
```

Duplications:

```
3-31  TGTGTGTGTGTGGGTGTGGTGTGGTGTGTGGTGTGGTGTGTGTGTGTGTGGGTGTGGTGTGGTGTGTGG
3-138 TGTGTGTGTGTGTGGGTGTGGTGTGGTGTGTGGTGTGGTGTGTGGGTGTGTGGGTGTGGTGTGTGTGGTGTGT
3-376 TGTGTGTGTGTGGGTGTGGTGTGGTGTGTGGTGTGGTGTGTGGGTGTGTGGGTGTGGTGTGTGTGGTGTGT
1-42  TGTGTGTGTGTGGGTGTGGTGTGGTGTGTGGTGTGGTGTGTGGGTGTGTGGGTGTGGTGTGTGTGGTGTGT

3-138 GTGGGTGTGGGTGTGGTGTGGGTGTGGTGTGTG
3-376 GGGTGTGGGTGTGGTGTGGGTGTGGGTGTGGTGTGTGGGTGTGTGGGTGTGGTGTGTGTGGTGTGTGGGTG
1-42  GGGTGTGGGTGTGGTGTGGGTGTGGGTGTGGTGTGTGGGTGTGGGTGTGGTGTGGGTGTGGGTGTGGTGTG

1-42  TGGGTGTGGTGTGGGTGTGGTGTGGGTGTGGTGTGG
```

Multiple Insertions & Deletions:

```
3-255 TGTGTGTGTGTGGGTGTGGTGTGGTGTGTGG-----------------------------GTGTGCGG
3-49  TGCGTGTGTGTGGGTGTGGTGTGGTGTGTGGTGTGGTGTGTGGGTGTGTGGGTGTGGTGTGTGTGGTGTGT
3-116 TGTGTGTGTGTGGGTGTGGTGTGGTGTGTGGTGTGGTGTGTGGGTGTGTGGGTGTGGTGTGTGTGGTGTG-
3-357 TGTGTGTGTGTGGGTGTGGTGTGGTGTGTGGTGTGGTGTGTGGGTGTGTGGGTGTGGTGCGTGTGGTGTGT
3-254 TGTGTGTGTGTGGGTGTGGTGTGGTGTGTGGTGTG-----TGGGTGTGTGGGTGTGGTGTGTGTGGTGTGT
3-17  TGTGTGTGTGTGGGTGTGGTGTGGTGTGTGGTGTGGTGTGTGGGTGTGTGGGTGTGGTGTGTGTGGTGTGT
1-42  TGTGTGTGTGTGGGTGTGGTGTGGTGTGTGGTGTGGTGTGTGGGTGTGTGGGTGTGGTGTGTGTGGTGTGT

3-49  GGGTGTGGGTGTGGTGTGGGTGCGGGTGTG-----TGGGTGTG
3-116 -------------------GGTGTGGGTGTGGTGTGTGGGTGTGG
3-357 GGGTGCGGGTGTGGTGTGGGTGTGGTGTGTGTGGTGTGTGGGTGTGGGTGTGGTGTGGG
3-254 GGGTGTGGGTGTGGTGTGGGTGTGGGTGTGGTGTGTGGGTGTGGGTGTGGTGTGGGTGTGGG
3-17  GGGTGTGGGTGTGGTGTGGGTGTGG------TGTGTGGGTGTGGGTGTGGTGTGGGTGTGGGTGTGG
1-42  GGGTGTGGGTGTGGTGTGGGTGTGGGTGTGGTGTGTGGGTGTGGGTGTGGTGTGGGTGTGGGTGTGGTGTG
```

Unique Recombinant Sequence:

```
3-184 TGTGTGTGTGTGGGTGTGGTGTGGGTGTGGTGTGGTGTGTGGTGTGGTGTGTGGGTGTGTGGGTGTGGTGT
1-42  TGTGTGTGTGTGGGTGTGGTGTGGTGTGTGGTGTGGTGTGTGGGTGTGTGGGTGTGGTGTGTGTGGTGTGT

3-184 GTGTGGTGTGTGGGTGTGGGTGTGG
1-42  GGGTGTGGGTGTGGTGTGGGTGTGGGTGTGGTGTGTGGGTGTGGGTGTGGTGTGGGTGTGGGTGTGGTGTG

1-42  TGGGTGTGGTGTGGGTGTGGTGTGGGTGTGGTGTGG
```

NOTES:

Sequence 3-184 aligns with the reference sequence either with a 11-nt insertion, or it contains a duplication event where the recombinant sequence highlighted in both grey and black) aligns with the boxed sequence in 1-42.

Supplemental References

1. Dandjinou, A. T., Larrive, M., Wellinger, R. E. & Wellinger, R. J. (2006) *Methods Mol Biol* **313,** 193-208.
2. Johnson, F. B., Marciniak, R. A., McVey, M., Stewart, S. A., Hahn, W. C. & Guarente, L. (2001) *Embo J* **20,** 905-13.
3. Ivessa, A. S., Zhou, J. Q., Schulz, V. P., Monson, E. K. & Zakian, V. A. (2002) *Genes Dev* **16,** 1383-96.
4. Bystricky, K., Laroche, T., van Houwe, G., Blaszczyk, M. & Gasser, S. M. (2005) *J Cell Biol* **168,** 375-87.
